# The Unrecognized Death Toll of COVID-19 in the United States

**DOI:** 10.1016/j.lana.2021.100033

**Published:** 2021-08-10

**Authors:** Seyed M. Moghadas, Alison P. Galvani

**Affiliations:** 1 Agent-Based Modelling Laboratory, York University, Toronto, Ontario, Canada M3J 1P3; 2 Center for Infectious Disease Modeling and Analysis (CIDMA), Yale School of Public Health, New Haven, Connecticut 06520, USA

Since the identification of SARS-CoV-2 in December 2019, the virus has exacted a devastating toll on global health. In the United States (US), the COVID-19 pandemic has caused more than 33 million confirmed infections and over 600,000 reported deaths as of July 1, 2021. However, recent estimates of undiagnosed infections [1] and under-reported deaths [2], [3], [4] demonstrate that the true burden of COVID-19 in the US, and likely in other countries, has not been fully captured.

In *The Lancet Regional Health* – *Americas*, Iuliano et al [4] used an excess-mortality Poisson regression model to estimate the number of deaths attributable to COVID-19 in the US from March 2020 to May 2021. By adjusting all-cause death counts for incomplete reporting, Iuliano et al [4] fitted their age-stratified model to each American state. All-cause excess mortality includes deaths both from the virus itself and those that arise from the externalities of the pandemic. The excess deaths metric thereby includes deaths for which COVID-19 was not necessarily the proximate cause, but where an overtaxed healthcare system led to failures in addressing other causes of mortality.

Nationally, Iuliano et al [4] estimated a 24% rate in under-reporting, meaning that over 180,000 additional deaths were either directly or indirectly attributable to COVID-19 beyond the 582,135 that were reported on death certificates by May 2021. The majority of unrecognized deaths were estimated to have occurred during the early months of pandemic spread when the healthcare system was overwhelmed and testing inadequate.

Implementation of testing in the US was considerably slower than in other countries. A week following confirmation of the first hundred COVID-19 cases, the US had conducted a total of 0.035 tests per 1000 capita. [5] At this same milestone, countries in the European Union ranged between 0.17 and 17.69 tests per 1000 capita. [5] Initial tests distributed by the US Centers for Disease Control and Prevention (CDC) were faulty. [6] While testing gradually expanded, it was nonetheless outpaced by the rapid growth of the outbreak during the first pandemic wave in the US. The mismatch between diagnostic capacity and incidence may have suppressed reporting rates even as the number of tests produced increased, particularly during local surges in cases.

As a result of the delay in testing availability, there was a prolonged period during which many US COVID-19 deaths and cases were not identified. Furthermore, a COVID-19 death had been retrospectively confirmed on February 6, 2020, much earlier than the first death contemporaneously reported in Seattle on February 29. [7] In addition, the presence of SARS-CoV-2 antibodies from blood samples drawn during the week of February 23, 2020 in New York City suggests that the virus was circulating there much earlier than March 1, the date that the first COVID-19 case was reported in the city. [8] Collectively, this evidence suggests that the lag between COVID-19 spread and scale-up of testing was even longer than indicated by confirmed cases alone.

Without testing, COVID-19 deaths are not included in the confirmed counts. To take into account the impact of such under-reporting, the model by Iuliano et al [4] incorporates a state-specific covariate that represents the weekly positivity rate of the total SARS-CoV-2 tests administered. As more tests are administered relative to the number of cases, the positivity rate falls. Consequently, this rate may be considered as an inverse indicator for completeness in reporting.

The lack of universal healthcare coverage in the US poses challenges during a pandemic unique among high-income countries. Prior to emergency federal appropriations, concern about medical bills likely deterred people from seeking diagnosis and treatment, simultaneously exacerbating both mortality and under-ascertainment. [9] In addition, when healthcare systems are overwhelmed and nosocomial COVID-19 transmission is rampant, people may be hesitant to seek medical attention. Such barriers to healthcare during the pandemic contribute to excess mortality beyond COVID-19 case fatalities for myriad conditions that can be life-threatening if care is not sought promptly.

The misattribution of COVID-19 mortality to other causes compounded discrepancies between reported and actual COVID-19 death counts. For example, COVID-19 can trigger cardiac arrest even in the absence of other symptoms. [10] Misattribution would have been particularly widespread early in the pandemic when our understanding of the clinical manifestations of COVID-19 was nascent.

Soberingly, the results of Iuliano et al [4] indicate that the COVID-19 outbreak in the US has been even more devastating to public health than is indicated by official reports. Accurate estimates of mortality burdens attributable to a disease are fundamental to decision-making regarding mitigation strategies and optimal resource allocation. Under-estimation of the COVID-19 death also impacts the willingness of the public to adhere to public health recommendations, including vaccination and non-pharmaceutical interventions, that are instrumental to controlling the pandemic.

## Contributors

Both the authors contributed equally to the article.

## Declaration of Competing Interests

None.
